# HNF4α isoforms: the fraternal twin master regulators of liver function

**DOI:** 10.3389/fendo.2023.1226173

**Published:** 2023-08-03

**Authors:** Sarah H. Radi, Kiranmayi Vemuri, Jose Martinez-Lomeli, Frances M. Sladek

**Affiliations:** ^1^ Department of Biochemistry, University of California, Riverside, Riverside, CA, United States; ^2^ Department of Genetics, Human Genetics Institute of New Jersey, Rutgers, The State University of New Jersey, Piscataway, NJ, United States; ^3^ Cancer Institute of New Jersey, Rutgers, The State University of New Jersey, New Brunswick, NJ, United States; ^4^ Department of Molecular, Cell and Systems Biology, University of California, Riverside, Riverside, CA, United States

**Keywords:** HNF4α, structure, isoforms, metabolism, liver

## Abstract

In the more than 30 years since the purification and cloning of Hepatocyte Nuclear Factor 4 (HNF4α), considerable insight into its role in liver function has been gleaned from its target genes and mouse experiments. HNF4α plays a key role in lipid and glucose metabolism and intersects with not just diabetes and circadian rhythms but also with liver cancer, although much remains to be elucidated about those interactions. Similarly, while we are beginning to elucidate the role of the isoforms expressed from its two promoters, we know little about the alternatively spliced variants in other portions of the protein and their impact on the 1000-plus HNF4α target genes. This review will address how HNF4α came to be called the master regulator of liver-specific gene expression with a focus on its role in basic metabolism, the contributions of the various isoforms and the intriguing intersection with the circadian clock.

## 1 Introduction

Whether referred to as “the seat of the soul” as the ancient Babylonians believed, “the seat of our darkest emotions” as Plato postulated, a term of endearment in Urdu, or the literal translation of “courage” in the Zulu language, one thing is clear – almost every culture past and present recognizes the importance and uniqueness of the liver. This review will address key aspects of the transcription factor HNF4α which is considered to be the master regulator of liver-specific gene expression, including the role of its two promoters and the alternatively spliced isoforms they regulate. Furthermore, while mutations in the human *HNF4A* gene and/or its target genes have been associated with several diseases, including hemophilia (1), inflammatory bowel disease (IBD) (2) and various cancers, including hepatocellular, colorectal, renal, and gastric carcinomas (3–5), this review will focus on its role in carbohydrate and lipid metabolism in the liver.

In 1996, *HNF4A* was identified as the gene mutated in Maturity Onset Diabetes of the Young 1 (MODY1), an inherited form of type 2 diabetes that causes diabetes in patients in young adulthood (6). Patients are born with hyperinsulinemia and increased body size, but it is not until they are older that their pancreas fails to secrete insulin in response to elevated blood glucose (7). These clinical findings highlighted the role of HNF4α in glucose metabolism (and insulin secretion) but many questions remain about the MODY1 mutations and the precise role of the different HNF4α isoforms in basic metabolism (8–11).

## 2 Liver structure and metabolic functions

The liver is the largest internal and main metabolic organ in the body. It is critical for nearly all bodily functions as it provides energy during periods of fasting/starvation, maintains homeostasis between meals and stores excess lipids and carbohydrates postprandially. Post-prandial nutrients and other chemical compounds, including glucose, lipids, amino acids and xenobiotics, make their way from the intestine directly to the liver through the hepatic portal system. The liver absorbs, packages, detoxifies, metabolizes and distributes these compounds to all the other tissues via the circulatory system.

The liver is composed of several lobes consisting primarily of hepatocytes, sinusoidal endothelial cells, stellate cells and Kupffer cells. Hepatocytes, which carry out the metabolic functions of the liver, are the predominant cell type in the liver (~70% by cell number) – this relative homogeneity, as well as the sheer size and accessibility of the liver, made it the ideal organ for early studies on tissue-specific gene expression (12).

**Figure 1:**
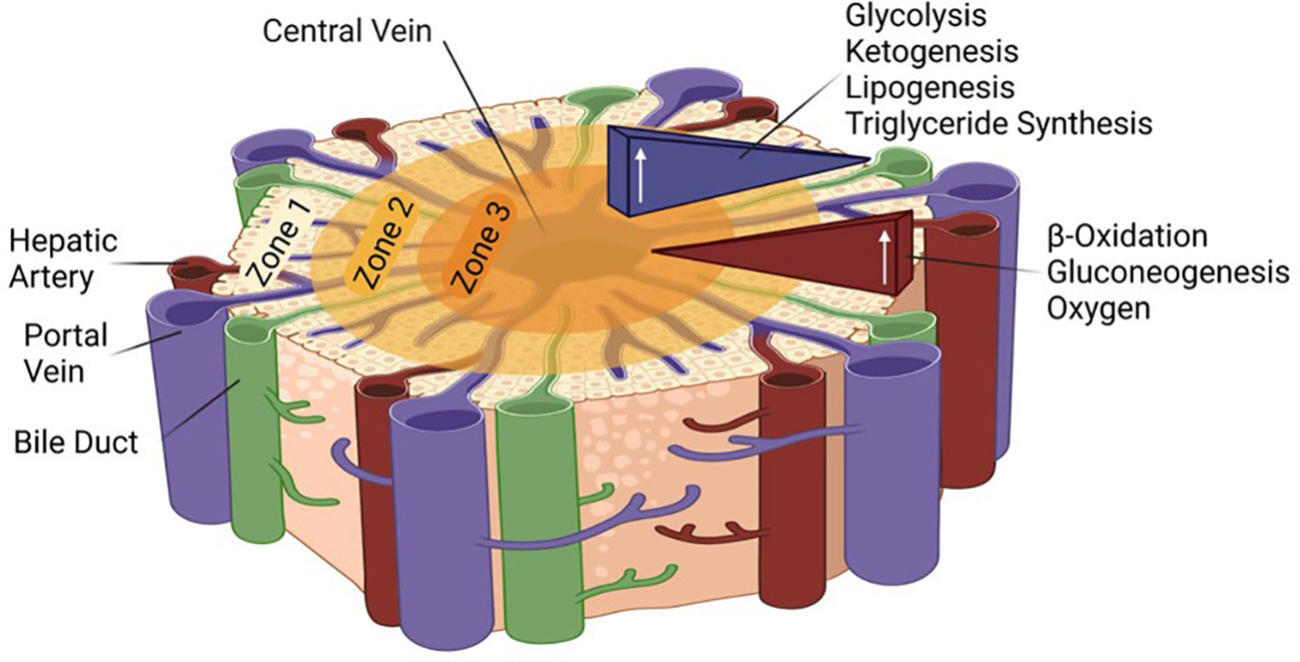
Zonation in the liver lobule and associated metabolic pathways. The role of the different HNF4α isoforms in the different zones remains to be determined.

Due to large metabolic demands of the body and the essential functions of the liver, the tissue is extremely well vascularized and uses 25% of the cardiac output although it makes up only about 2.5% of the total body weight (13, 14). Nonetheless, oxygen is not maintained at a constant pressure throughout the tissue; it differs based on the proximity to the portal triad, which includes the portal vein, hepatic artery, and bile duct. While it is assumed that all hepatocytes are capable of the same functions, the liver is able to compartmentalize and focus certain functions via this oxygen gradient. Each lobe of the liver consists of three zones with zone one receiving the most oxygen and having the highest respiratory enzyme activity, including beta-oxidation and gluconeogenesis. Closest to the central vein, zone three is the least oxygenated and carries out glycolysis, lipogenesis, and ketogenesis (15) (**Figure 1**). It has been proposed that all zones play a role in liver homeostasis and regeneration (16, 17). Finally, the liver, which synthesizes bile from bilirubin, bile salts, and cholesterol to aid in fat digestion, surrounds the gallbladder, the site of bile storage.

The liver maintains glucose homeostasis between meals via release of stored glycogen and gluconeogenesis. The liver can initiate *de novo* glucose synthesis from lactate, pyruvate, oxaloacetate and/or glucogenic amino acids. Once produced, new glucose is transported through the blood to supply the brain, the muscles, and other organs with energy.

Gluconeogenesis is an energetically expensive process that is heavily regulated by hormones, such as insulin and glucagon, as well as by enzymes whose expression is regulated by various transcription factors. Gluconeogenesis in the liver is also dependent on the availability of oxaloacetate. If gluconeogenesis continues for an extended period of time, oxaloacetate levels will be depleted, and gluconeogenesis and the tricarboxylic acid (TCA) cycle will shut down. This causes the liver to switch to metabolism of fat to supply the body with energy. During starvation as well as prolonged fasting, fatty acids are broken down to produce acetyl-CoA.

During ketogenesis the liver converts acetyl-CoA into ketone bodies such as β-hydroxybutyrate, which are secreted into the bloodstream where they are transported to other organs as an energy source. This process of hepatocyte-driven ketogenesis is absolutely critical for the brain to continue to function during periods of fasting/starvation: not only can the brain not carry out gluconeogenesis, it also cannot utilize fat as an energy source. Indeed, during hypoglycemia, up to two-thirds of the energy needs of the brain can be provided by ketone bodies produced in the liver. Similar to gluconeogenesis, ketogenesis is also tightly controlled by insulin, glucagon, and various transcription factors.

In addition to the gluconeogenic and ketogenic pathways, the liver is the main site for fatty acid synthesis and distribution. The liver also carries out *de novo* fatty acid synthesis from excess short chain fatty acids, carbohydrates and/or proteins. The liver can store the synthesized fat in lipid droplets, the excess of which causes non-alcoholic fatty liver disease (NAFLD). Obesity and type 2 diabetes are the most common risk factors that lead to NAFLD, which is increasing in incidence in the United States and worldwide (18). It is estimated that one-third of adults worldwide have fatty liver, and it is not always associated with obesity or alcohol (19, 20). Non-alcoholic steatohepatitis can also lead to cirrhosis and liver cancer and ultimately liver failure.

**Figure 2:**
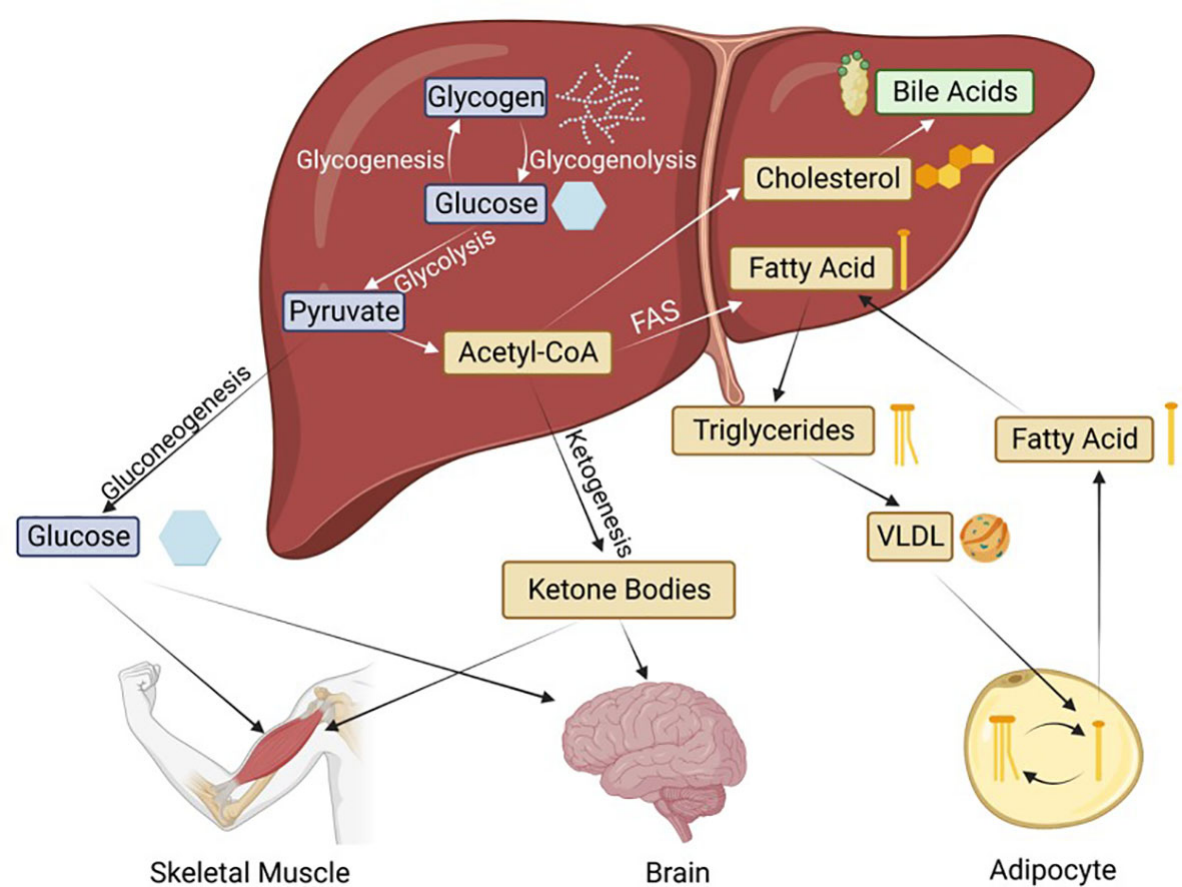
Basic metabolic pathways in the liver and the transport to peripheral tissues. Hepatic HNF4α is known to regulate genes involved in all of these processes. FAS, Fatty acid synthesis; VLDL, very low-density lipoprotein. Blue boxes indicate glucose metabolism intermediates, thought to be regulated by the P1 isoforms; while yellow and green are the lipid metabolism intermediates, thought to be regulated by the P2 isoforms.

A depiction of the intersection of these metabolic pathways is shown in **Figure 2**. We now know that HNF4α is critical to all of these metabolic processes. Loss of HNF4α expression is associated with liver cirrhosis and reintroduction of HNF4α can reverse cirrhosis (21), underscoring the essential nature of this transcription factor to overall liver function.

## 3 Hepatocyte nuclear factors

The hepatocyte nuclear factors (HNFs) constitute a group of transcription factors which control gene expression and development in various tissues. While they were originally identified in the liver (22–24), it was quickly found that they exhibit differential gene expression patterns across several tissues of the gastrointestinal system (25). HNF1 (POU HOMEO), HNF3 (FOXO), HNF4 and HNF6 (WINGED HELIX), belong to different transcription factor families. HNF4, the topic of this review and by far the most abundant HNF in the liver, is a member of the nuclear receptor superfamily (24) and regulator of *HNF1A* (26). In mammals, HNF4 is encoded by two distinct genes – *HNF4A and HNF4G*, located on human chromosomes 20 and 8, respectively (27). Another variant, HNF4β is expressed in *Xenopus laevis*, where it shares binding sites with HNF4α but is a less potent transactivator (28). HNF4γ has two splice variants – HNF4γ1 is expressed in the kidney, intestine, colon and pancreas whereas HNF4γ2 is an intestine-restricted isoform (29).

## 4 HNF4α forms combinatorial heterodimers among itself and with HNF4γ

Despite extensive investigation into potential dimerization between HNF4α and other nuclear receptors, particularly the promiscuous retinoid X receptor RXR, no credible evidence of heterodimer formation with other nuclear receptors has been reported. Furthermore, amino acid residues in HNF4α have been identified that would prevent such heterodimerization (30–32). The exception is HNF4γ which contains the same critical residues as HNF4α and has been shown to heterodimerize with it (33). HNF4α also forms transcriptionally active heterodimers among its twelve isoforms generated by alternative promoter usage and splicing. There are examples of both the homo- and heterodimers of HNF4α regulating their own subset of target genes with varying levels of transcriptional efficiency. Individual isoforms co-expressed in cells revealed pairings such as HNF4α3+α8 and HNF4α2+α3, which exhibit substantial differences in their transcriptional activity relative to the corresponding homodimers. HNF4α3+α8 activates expression of two of its targets, *CYP7A1* and *ALDOB*, at much higher levels than HNF4α3 or HNF4α8 alone. Similarly, HNF4α2+α3 acts as a “loss of function” heterodimer which downregulates the same target gene subset relative to HNF4α2 or HNF4α3 homodimers (34). These findings highlight the need for a more comprehensive study of the transcriptional profile of the various HNF4α isoforms as functions previously ascribed to HNF4α homodimers could potentially also be attributed to heterodimers of HNF4α/γ or the HNF4α isoforms.

## 5 HNF4α is required for early development of the liver and pancreas

HNF4α is first detected in the visceral endoderm at embryonic day E4.5 in the mouse. Homozygous deletion of HNF4α in the germline is lethal at E4.5, due to defects in the visceral endoderm which prevent gastrulation (35, 36). Complementation of HNF4α-deficient embryos with a tetraploid, embryo-derived, wild-type visceral endoderm rescues this early developmental lethality and the embryos gestate normally, underscoring the importance of HNF4α for early development (37). Subsequently, HNF4α is found in the liver bud from E8.5 onwards, showing early commitment towards the hepatoblast lineage and a role in the epithelial transformation of the developing liver (38, 39). Ablation of HNF4α in murine fetal livers blocks hepatocyte differentiation and proper formation of hepatic epithelium and sinusoidal endothelium (37, 40). Accessory transcription factors HNF1β, GATA-6, OC-1 and FOXA2 all coordinate with HNF4α to modulate the varying stages of liver development (41).

During the course of pancreatic development, HNF4α expression is detected in most epithelial cells of the pancreatic bud from E9.5 while in the adult it is more restricted to islet cells (42). By the onset of maturity, HNF4α is expressed primarily in the liver, kidney and intestines although it still plays a role in the pancreas, as evidenced by mutations in the *HNF4A* gene in MODY1 patients (6). It should be noted that there are differences in the HNF4α isoforms expressed during development of the two organs, with P1-derived HNF4α1-6 predominant in the liver, and P2-derived HNF4α7-12 predominant in the early pancreas (34, 43, 44).

## 6 Molecular structure of HNF4α

As a member of the nuclear receptor superfamily of ligand-dependent transcription factors (NR2A1), HNF4α is comprised of five distinct structural domains. The ~200 amino acid ligand binding domain (LBD) that defines the nuclear receptors contains a hydrophobic pocket that binds ligands and facilitates the transactivation of genes. Ligand binding induces conformational changes in the LBD which allows it to interact with a signature LXXLL motif in transcriptional co-activators (45) or a LXXXIXXX(I/L) motif in transcriptional co-repressors (46). However, despite being a nuclear receptor, HNF4α is constitutively active and does not require the binding of a ligand to mediate gene activation (47). The LBD of HNF4α crystallizes as a canonical homodimer with intermolecular salt bridges and hydrogen bonds contributing to the stability of the interface (30, 31, 48–50).

The LBD is connected to a highly conserved DNA binding domain (DBD) by a hinge region which facilitates free rotation between the two domains and contains the nuclear localization signal (NLS) (51). The DBD, comprised of two cysteine-rich zinc finger motifs, dimerizes on the DNA even in the absence of the LBD (48, 52, 53). DNA binding induces a conformational change in the LBD of HNF4α, revealing another dimerization interface and leading to an increase in the overall stability of the HNF4-DNA complex, a process essential for mediating its transcriptional activity (52, 53). The DBD and the LBD are bordered by two transactivation domains – AF-1 and AF-2, respectively. The 24 amino acids in the AF-1 act as a constitutive, autonomous transactivator domain, with Tyr6, Tyr14, Phe19, Lys10, and Lys17 essential for AF-1 activity (54, 55). The AF-2 domain in the C-terminal end of the LBD interacts with co-activators or other transcription factors (56). Unlike AF-1, the activity of the AF-2 domain depends on ligand binding in the LBD in most nuclear receptors, although evidently not HNF4α (47). Somewhat unique to HNF4 is the presence of a large domain (F) at the C-terminus which represses transactivation; it contains a proline-rich region that plays a role in distinguishing between transcriptional co-activators and co-repressors in a ligand-independent fashion (57, 58). Additionally, a 10-amino acid insertion in the F domain introduced by alternative splicing modulates the repressive activity of the F domain (56). *HNF4A* is extensively modified post-translationally, including through phosphorylation and acetylation (59). These modifications are discussed in other review articles in this Special Topics edition.

More than 100 mutations in *HNF4A* have been associated with MODY1. The vast majority are in either the DBD or LBD with just a couple in the N-terminal region and none in the F domain (10). The first MODY1 mutation identified was Q268X in the middle of the LBD (6, 60). Since this mutation truncates the protein before the salt bridge that prevents heterodimerization with other nuclear receptors, there was a possibility of a dominant negative effect of this MODY1 mutation that could have impacted many other nuclear receptor pathways. Fortuitously, the mutant HNF4α protein was localized to the nuclear membrane and thus inaccessible to other transcription factors (61). There are several other nonsense mutations in MODY1 patients although the majority of the MODY1 mutations are missense mutations (10) raising the possibility of more subtle alterations in specific HNF4α functions.

## 7 HNF4α: master regulator of liver-specific gene expression

Since its initial identification, HNF4α has been implicated in the regulation of hepatic lipid metabolism. Indeed, a liver-specific response element in the human apolipoprotein CIII (*APOC3*) gene was used to clone the first HNF4 cDNA from rat liver (24) while *APOA1* and *APOB* (which encode protein components of HDL and LDL, respectively) were early HNF4α target genes (62, 63). Similarly, key genes involved in glucose metabolism, such as PEPCK (*PCK1*) and L-pyruvate kinase (*PKLR*), were also early targets of HNF4α (64, 65), even before MODY1 was associated with the *HNF4A* gene (6).

Using classical promoter-bashing approaches, the number of HNF4α binding sites in target gene promoters grew quickly. By the time the first draft of the human genome was released in 2001, there were more than 70 verified HNF4α binding sites in the literature. Early computational and wet bench approaches doubled the number of HNF4α binding motifs (66) while the advent of Chromatin Immunoprecipitation (ChIP) followed by genomic sequencing techniques (ChIP-chip, ChIPseq assays) identified hundreds more potential HNF4α target genes in liver and pancreas (67, 68). Since a ChIP signal is not necessarily due to direct binding to the genomic DNA and since identification of the exact sequence to which a transcription factor binds in a ChIP peak can be challenging, protein binding microarrays (PBMs) were used to accelerate the identification of HNF4α binding sites *in vitro*. Cross referencing of those sites with expression profiling of HepG2 cells with or without HNF4α led to the identification of 240 new direct HNF4α human target genes, including new functional categories of genes not typically associated with HNF4α, such as cell cycle, immune function, apoptosis, stress response, and cancer-related genes (69). It also earned HNF4α the title of master regulator of liver-specific gene expression, which has persisted to this day (70).

The PBM technology led to the identification of more than 20,000 different DNA sequences to which HNF4α binds as well as a binding motif unique to HNF4α (71, 72). This is important given that many of the “orphan” nuclear receptors like HNF4, COUP-TF and RXR share a common DNA binding motif consisting of a direct repeat of AGGTCA half sites (AGGTCAxAGGTCA). Indeed, competition for control of expression of liver-specific genes by HNF4α and other nuclear receptors was noted early on (73). The PBM studies also led to the identification of >60 unique, low affinity HNF4α binding sites located in more than a million Alu sequences which are unique to primate genomes; this raised the possibility of sequestration of HNF4α protein by binding repetitive genomic sequence as a novel mechanism by which to regulate HNF4α function (74). Fortunately, HNF4α is one of the most abundant transcription factors in the liver; the initial purification of HNF4α required only a 5000 to 10,000-fold enrichment (24). On the RNA level, HNF4α expression far surpasses that of all the other liver-enriched transcription factors (HNF1, C/EBP, HNF3, HNF6), all other nuclear receptors, and even TATA binding protein and RNA polymerase (75). As it turns out, the moniker of “master regulator of liver-specific gene expression” does indeed seem to be appropriate.

## 8 P1- vs. P2-HNF4α

**Figure 3:**
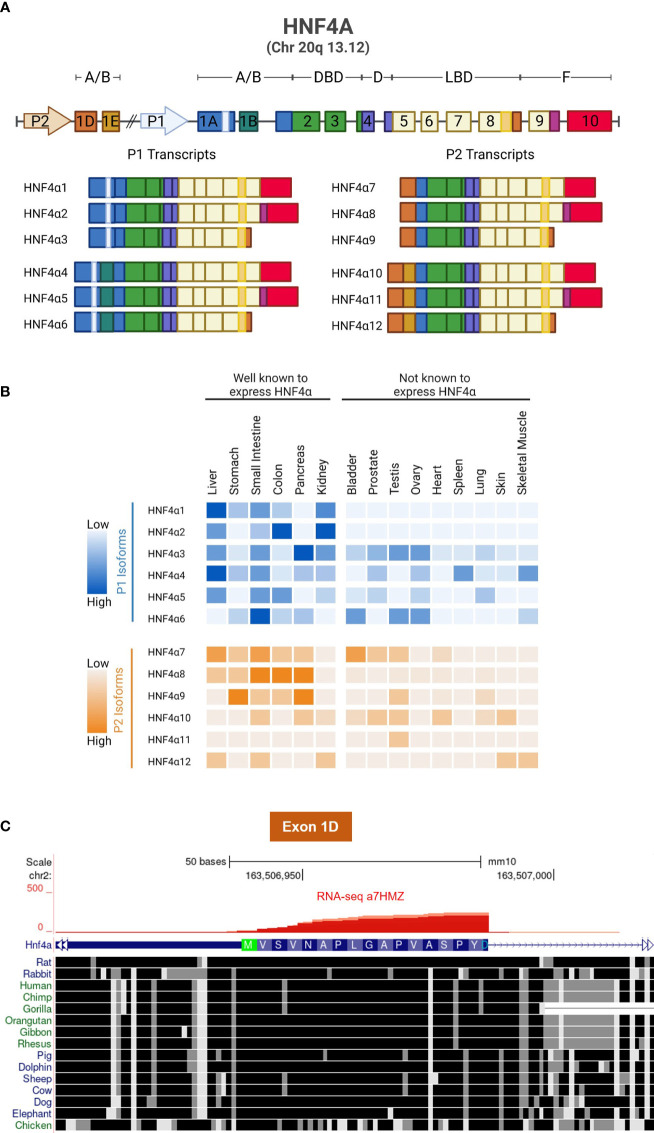
HNF4α isoforms and conservation of Exon 1D. **(A)** Schematic showing the gene structure of human HNF4A and its 12 transcripts, generated by alternative P1 and P2 promoters and alternative splicing in the N- and C-termini. **(B)** Relative mRNA expression of human HNF4α isoforms, data from 34. **(C)** UCSG Genome Browser view (Multiz Alignment and Conservation track enabled) of Exon 1D adjacent to the P2 promoter of mouse *Hnf4a* gene (mm10) showing conservation from dolphin to human with only a single amino acid differing in most species (Ala13). Even non-mammalian animals such as chicken exhibits considerable conservation in Exon 1D. RNAseq reads from α7HMZ male mice confirm the location of Exon 1D (**Figure 4** and 75).

Expression of the *HNF4A* gene is driven by two highly conserved promoters, denoted P1, which is closest to the gene body, and P2, which is ~50 kb upstream. Together they drive the expression of twelve different HNF4α transcript variants referred to as isoforms. P1 activation leads to expression of HNF4α1, HNF4α2, HNF4α3, HNF4α4, HNF4α5 and HNF4α6; while P2 activation leads to HNF4α7, HNF4α8, HNF4α9, HNF4α10, HNF4α11 and HNF4α12 (**Figure 3A**). The tissue distribution of the twelve isoforms has been characterized by PCR and can be divided into well-established HNF4α-expressing tissues and other tissues that have not been examined in detail for HNF4α expression or function (**Figure 3B**) (34). The first exon of the P2 promoter (exon 1D), like the rest of the *HNF4A* gene, is highly conserved across most vertebrates (**Figure 3C**).

Early studies investigating HNF4α did not explore the role of different isoforms and the majority of the studies in the adult liver focused solely on the role of the predominant P1-HNF4α, particularly HNF4α1, the first transcript cloned (24). Even though the P2-isoform was discovered in 1998 in an undifferentiated pluripotent embryonal carcinoma cell line (F9) (76), it took several years before any functional differences were observed in the P1 and P2 isoforms (77). More recently, the scientific community has taken an interest in exploring the differences between the HNF4α isoforms in all of the tissues where they are endogenously expressed (34). The small differences in protein size can sometimes be revealed using Western blot analysis, and antibodies specific to P1- and P2-HNF4α are commercially available (3, 78).

The most well characterized and most abundantly expressed isoforms are HNF4α1/2 and HNF4α7/8, which differ in the N-terminal AF-1 domain that interacts with co-activators (56, 58, 77, 79). The P1-HNF4α isoforms contain exon 1A while the P2 isoforms contain exon 1D. The difference between HNF4α1 and HNF4α2 (and HNF4α7 and HNF4α8) is that the latter has the ten amino acid insert in the F-domain which modulates the transcriptional activity of HNF4α (58). The remaining domains – DBD, LBD, and hinge region – are identical in all isoforms. (Since the isoforms are very similar, though not identical, we chose the comparison to fraternal twins in the title.)

**Figure 4:**
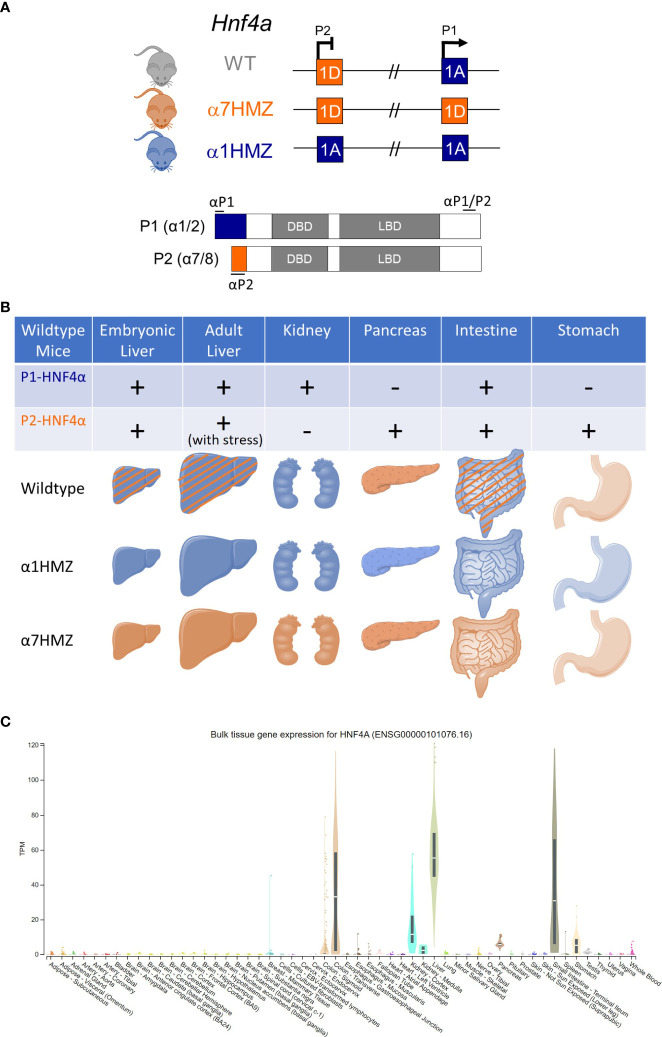
HNF4α exon swap mice and expression of P1- and P2-HNF4a isoforms in different tissues. **(A)** *Hnf4a* locus in wildtype (WT) and exon swap mice (α7HMZ and α1HMZ); HNF4A protein structure with isoform-specific antibodies noted (αP1, αP2, αP1/P2). DBD, DNA binding domain; LBD, ligand binding domain. **(B)** Depiction of the HNF4α isoforms expressed in different tissues in WT, α1HMZ and α7HMZ mice. Stripes indicate both isoforms are present. **(C)** Bulk tissue expression in adult humans (males and females) for HNF4A from The Genotype-Tissue Expression (GTEx) Project. Aside, from liver, intestines and pancreas discussed in the text, expression of HNF4α in the various cell types of these different tissues has not been exhaustively examined.

Among the 54 non-diseased human tissues in the Genotype-Tissue Expression (GTEx) Project, bulk RNAseq data shows that HNF4α is selectively expressed in a few different tissues in the adult, with the greatest expression in liver followed by large (colon) and small intestines and then kidney and finally pancreas and stomach (**Figure 4C**). Both P1- and P2-driven HNF4α isoforms are expressed in the fetal liver, although after birth the expression of P2-HNF4α decreases dramatically and the expression of P1-HNF4α increases (80) (**Figure 4B**). Initially, it was thought that P2-HNF4α was not expressed in the normal adult liver due to repression of the P2-promoter by P1-HNF4α (80). However, we and others have observed P2-HNF4α expression in the adult liver at different times of the day as well as in response to fasting, high fat diet and alcoholic fatty liver (75, 81–83). Furthermore, P2-HNF4α expression often increases in liver cancer as P1-HNF4α expression decreases (3, 84). These and other findings lead to the dogma that P1-HNF4α acts as a tumor suppressor in the liver while P2-HNF4α is at least permissive of proliferation, both of which were found to be the case in colon cancer (78, 85).

P1-HNF4α is the predominant form expressed in the proximal tubules of the kidneys, though its precise role in that tissue remains to be determined (**Figure 4B**); some have speculated that it could play a role in gluconeogenesis in that tissue (86, 87). P2-HNF4α is the predominant form in the pancreas where it plays a role in insulin secretion from β-cells (42, 44, 88). P2-HNF4α is also the predominant form in the stomach, though P1-HNF4α seems to be found there as well; both isoforms apparently play a role in differentiation of the epithelial cells as well as the development of gastric cancer (89). Both P1- and P2-driven HNF4α are expressed throughout the small intestines and the colon although anecdotal evidence suggests a relative increase in expression of P2-HNF4α as the intestinal tract progresses from the duodenum to the colon. In the distal colon, P1-HNF4α is expressed at the top of the colonic crypts in the differentiated portion, while P2-HNF4α is expressed in the bottom half of the crypts in the proliferative compartment (78). Expression of HNF4α in the intestines and colon is relevant given that *HNF4A* is an IBD susceptibility gene (2) and P1-HNF4α (but not P2-HNF4α) is a target of Src tyrosine kinase in human colon cancer (90, 91).

## 9 *Hnf4a* exon swap mice

**Figure 5:**
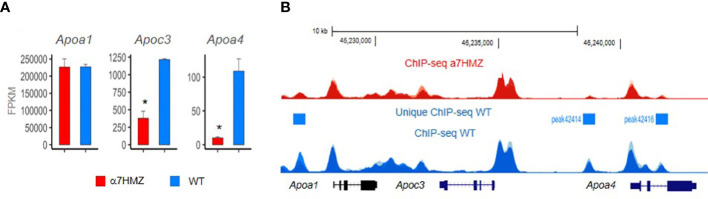
HNF4a isoform-specific targets in apolipoprotein locus in mouse chromosome 9. The region contains multiple HNF4a binding sites which have been shown *in vitro* to regulate expression of multiple Apo genes, including one of the first HNF4a target genes – the Apoc3. RNAseq **(A)** and ChIPseq **(B)** from biological replicates of livers from 3 adult male mice at ZT3.5 fed a standard vivarium chow diet – wildtype (WT) expresses only P1-HNF4a and a7HMZ exon swap mice express only P2-HNF4a (75). **(A)** Average FPKM of RNAseq. * p-adj < 0.000001. **(B)** ChIPseq peaks visualized in UCSC Genome Browser mm10; y-axes are identical in WT and a7HMZ tracks. Peaks unique to WT called by MACS2 are indicated: they could explain the greater level of expression of Apoc3 and Apoa4 in WT livers, in addition to differential interactions between the HNF4a isoforms and other transcription factors (95).

Since the whole-body HNF4α knockout is embryonic lethal and a liver-specific knockout results in death at six weeks of age due to dyslipidemia, high serum bile acid levels and ureagenesis defects (35, 92, 93), an HNF4α exon swap mouse model was developed to examine the effects of the HNF4α isoforms *in vivo* (94) (**Figure 4A**). The model allows examination of a single group of HNF4α isoforms, either P1- or P2-HNF4α, using physiological levels of expression since the promoter regions are not altered. In α7HMZ mice, exon 1A adjacent to the P1 promoter is replaced by exon 1D which is normally adjacent to the P2 promoter, creating mice that express only HNF4α protein with the N-terminal domain of P2-HNF4α (e.g., HNF4α7, HNF4α8, etc.) in all HNF4α-expressing tissues. In contrast, the reciprocal swap of exon 1D for exon 1A generates mice that express only P1-HNF4α proteins in α1HMZ mice. Both the α1HMZ and α7HMZ mice are fertile and viable, unless they are subjected to various conditions of stress. The α7HMZ mice have significantly lower levels of cholesterol, triglycerides, and free-fatty acids compared to wildtype and α1HMZ mice, but significantly higher levels of ketone bodies. They also have fattier livers under conditions of fasting which could be due to decreased expression of apolipoproteins that export fat from the liver to the other tissues (94) (**Figure 5A**). This initial characterization of the exon swap mice was the first indication that the different HNF4α isoforms may play different roles in basic metabolism. Subsequent studies by our group confirmed that livers from α7HMZ male mice exhibit a metabolic transcriptome, rather than one specific to liver cancer (75).

In addition to HNF4α, there are other transcription factors in the liver, especially nuclear receptors, that regulate genes involved in basic metabolism. These include: the glucocorticoid receptor (NR3C1, GR) which stimulates gluconeogenesis via interaction with HNF4α on the *PCK1* promoter and regulates the HNF4α promoter (65, 96, 97); the peroxisome proliferator activated receptors (NR1C, PPARs) which play critical roles in beta-oxidation of fatty acids and ketogenesis and, like HNF4α, have fatty acids as their ligands (97, 98); and the farnesoid X receptor (FXR, NR1H4) which regulates bile acid production and co-occupies many genes with HNF4α in the liver (99). The nature of these interactions on the molecular level and the specificity, if any, with respect to the HNF4α isoforms requires further investigation.

## 10 Mechanisms of HNF4A promoter switching

While the P1 and P2 promoters that drive the expression of the HNF4α isoforms have been dissected for relevant regulatory elements (80, 100), a mechanism for a potential switch between the promoters in the liver is less well defined. Emerging data suggest that antisense transcripts and DNA methylation may be involved. Antisense transcripts are a class of long, single-stranded non-coding RNAs which have been shown to be widespread in mammalian genomes and act as regulatory switches in gene expression circuits (101). In humans, HNF4α‐AS1(NR_109949.1) is a 648 nucleotide, antisense RNA located between the P1 and P2 promoters which makes it ideal for playing a role in the regulation of promoter switching; tellingly, it has also been implicated as a biomarker in hepatocellular carcinoma (HCC) and Crohn’s disease (102, 103). ChIP-seq data in mouse liver reveals P1‐HNF4α binding in the vicinity of the HNF4α‐AS1 promoter while luciferase assays show that P1- but not P2-HNF4α activates the HNF4α‐AS1 promoter (104). Moreover, HNF4α‐AS1 is primarily transcribed in the liver, kidney, and intestine where P1-HNF4α expression is predominant and P2-HNF4α expression is low (104) (**Figures 3B** and **4B**).

A recent study suggests that another noncoding RNA (H19) and DNA methylation may also be involved in the re-expression of P2-HNF4α in the adult liver (Da 83). H19 is one of several long ncRNA that regulate insulin signaling and glucose/lipid metabolism in various tissues (105). Da Li et al. found that fasting upregulates the H19 ncRNA in the liver, which subsequently increases HNF4α, PGC1α, PEPCK, and G6PC mRNA, and, unexpectedly, TET3 mRNA. TET3 is a DNA demethylase which increases expression of *Pck1*, *G6pc*, and glucose production. Increases in H19 and TET3 mRNA have been observed in human livers of type-2 diabetes patients, suggesting that this mechanism is likely conserved between humans and mice (Da 83)

Importantly, conditions that increased *Tet3* expression led to a specific increase in P2-HNF4α but not P1-HNF4α expression. Mice injected with P2-HNF4α specific shRNA adenoviral vector decreased fasting glucose, fasting insulin, *Pck1* and *G6pc* levels, and pyruvate tolerance (pyruvate tolerance tests are specific to gluconeogenic glucose production). Since TET3 is known to demethylate DNA and activate transcription, the authors speculate that it was demethylation of the P2-promoter that resulted in increased expression of P2-HNF4α and showed TET3 binding to the P2 promoter in association with FOXA2. In short, P2-HNF4α is increased in the livers of fasted mice, and leads to hepatic gluconeogenesis via activation of gluconeogenic genes such as *Pck1* and *G6pc* in conjunction with co-activator PGC1α (Da 83). While PGC1α was shown some time ago to be required for HNF4α activation of *Pck1* and *G6pc* in the fasted liver, a specific HNF4α isoform was not identified at that time (106, 107). This new study shows that PGC1α co-activates P2-HNF4α more effectively than P1-HNF4α on these gluconeogenic genes (Da 83).

## 11 Role of HNF4α in circadian rhythms and fasting

Daily fluctuations in physiological and behavioral processes rely on an intrinsic molecular clock and response to environmental changes (108). The intrinsic clock, or circadian rhythm, in mammals allows tissues to perform their designated function at specific times of the day and to anticipate changes from outside sources, thereby synchronizing mammalian physiology to the 24-hour solar day. Each tissue has its own peripheral clock, but they are all synchronized by the central molecular clock in the suprachiasmatic nucleus (SCN) in the brain. External cues that affect circadian behaviors are called “zeitgebers”. While the light/dark cycle is the most commonly studied zeitgeber, other zeitgebers include melatonin release and uptake, body temperature fluctuations, the feeding/fasting cycle and jet lag. Chronic jet lag induces spontaneous HCC in wild-type mice via a mechanism observed in obese humans involving nuclear receptor-controlled cholesterol and bile acid metabolism as well as xenobiotic metabolism pathways (109). Given the extensive role of HNF4α in basic metabolism in the liver, as well as liver cancer (3, 4), it is not surprising that HNF4α has been found to interact with proteins that regulate the circadian clock and play an active role in the hepatic circadian clock (84, 110, 111).

HNF4α represses the transcriptional activity of the essential circadian regulator CLOCK : BMAL1 (110). ChIP-seq analysis reveals co-occupancy of HNF4α and CLOCK : BMAL1 at many metabolic genes involved in lipid, glucose, and amino acid metabolism, creating a feedback loop in the liver-specific peripheral clock and impacting the circadian regulation of metabolic pathways. In short, HNF4α is essential for the circadian rhythmicity of liver (and colonic cells) where it is normally expressed and alters the intrinsic clock when it is ectopically expressed. Interestingly, HNF4α appears to inhibit the CLOCK : BMAL1 complex by a mechanism independent of CRY1, the canonical clock repressor. All of the HNF4A isoforms examined (HNF4α1, HNF4α2 and HNF4α8) caused this inhibition and the DBD, LBD and F domain were all required, suggesting that a common protein structure among the isoforms is responsible for the repression (110). In a follow up study, the authors show that HNF4A and BMAL1 reciprocally regulate each other’s genome-wide binding and that circadian rhythms are disturbed in *Hnf4a* knockout liver cells. The epigenetic state and accessibility of the liver genome dynamically changes throughout the day, synchronized with chromatin occupancy of HNF4A and clustered expression of circadian outputs (111).

The role of HNF4α in circadian regulation in liver cancer has also been examined. P2-HNF4α, which is often upregulated in liver cancer, is selectively induced in HCC, where it directly inhibits the expression of BMAL1 and leads to the cytoplasmic expression of the P1 isoform (84). Interestingly, induced expression of BMAL1 in HNF4α-positive liver cancer cells impairs growth in culture and *in vivo*. Manipulation of the circadian clock in HNF4α-positive HCC could be a strategy to slow or reverse growth of human HCC.

Finally, a study from our group in this issue of *Frontiers in Endocrinology* examines the effect of the P1- and P2-HNF4α isoforms on liver gene expression using the *Hnf4a* exon swap mice (75). We found that mice expressing only P2-HNF4α (α7HMZ) have elevated levels of ketone bodies upon fasting but do not survive a prolonged fast as well as mice expressing only P1-HNF4α (α1HMZ) or wildtype (WT) mice. Endogenous P2-HNF4α was expressed in the adult liver at ZT9 when levels of glucose are normally low and ketone body levels are high, an effect that was enhanced in CLOCK knockout mice. This is interesting when compared to P1- HNF4α, which does not oscillate as dramatically as the P2 isoform throughout the day, remaining at relatively stable levels in both wildtype and CLOCK knockout mice (75). PBMs revealed that P2-HNF4α and P1-HNF4α have essentially identical DNA binding specificity even in the context of liver nuclear extracts; P1-HNF4α also seems to have a preference for GC-rich motifs that bind SP1, consistent with interactions noted previously between these two transcription factors (95, 112). ChIPseq analysis also revealed very similar genome-wide binding of the P1 and P2 isoforms, despite a dysregulation of hundreds of genes, although there were some notable differences in chromatin binding in the *Apoa1 - Apoc3 - Apoa4* locus that correlated with levels of RNA expression (75) (**Figure 5**). In contrast, protein-protein interaction studies showed differential binding of HNF4α in wild-type livers compared to α7HMZ livers to several proteins, including those involved in the circadian clock (NFIL3, ARNTL, CLOCK) as well as nuclear receptors and other transcription factors (75). Those protein-protein interactions, as opposed to DNA specificity or access to the chromatin, are presumed to be responsible for the dysregulation of target genes in the livers of WT and α7HMZ livers, especially in the fasted state. Metabolomic analysis showed increased levels of lipids and ketone bodies in mice expressing only P2-HNF4α (α7HMZ); in contrast, levels of glucose, pyruvate and citric acid were lower in the α7HMZ mice (75), as noted previously (94). Finally, while the P2-HNF4α hepatic transcriptome was more similar to the fetal liver transcriptome than that of WT adult mice, it did not strongly resemble that of liver cancer and there was no increased incidence in liver tumors even in α7HMZ mice more than a year old (75). This suggests that while P2-HNF4α might be upregulated in human liver cancer, it does not appear to be a driver of the cancer phenotype, at least in mice.

## 12 The search for the HNF4 ligand

Crystallographic studies from two independent groups revealed a mixture of tightly bound fatty acids in the LBD of bacterially expressed HNF4α (49, 50). This led to the conclusion that HNF4α was not a druggable target as its ligand binding pocket was essentially permanently occupied (113). Given that bacterial cells are known to have different fatty acid compositions than mammalian cells, in order to identify HNF4α ligand(s) from a more physiologically relevant environment, HNF4α was immunoprecipitated from mouse liver and bound molecules were analyzed by gas chromatography/mass spectrometry (GC/MS) (47). The essential fatty acid linoleic acid (LA, 9, 12, octadecadienoic acid, C18:2, Δ^9,12^) was the only lipid found to be bound to endogenous HNF4α protein in mouse liver. Furthermore, when HNF4α was isolated from the livers of mice undergoing a prolonged fast, the amount of bound LA was noticeably decreased, consistent with depletion of LA during the fast. Follow up mutagenic studies in the ligand binding pocket confirmed specific binding while kinetic studies with isotopically labeled LA proved that binding was completely reversible (47). Expression profiling studies in the presence and absence of LA revealed that ligand binding only moderately affected the transcription of HNF4α target genes, an effect which could have been due to a decreased level of HNF4α protein in the presence of LA (47). Other nuclear receptor ligands are known to alter receptor stability, in addition to recruiting co-activators or co-repressors (114). Notably, HNF4α appears to have high endogenous transcriptional activity in its ligand-free state; its expression is also increased in the fasted state, due in part to the fact that insulin decreases the expression of P1-HNF4α via SREBPs (115). The role of the different HNF4α isoforms, if any, in terms of ligand function is not known: the LBDs of P1- and P2-HNF4α are identical but the AF-1 in other nuclear receptors is known to interact with the LBD and impact ligand function (79). There is one curious finding related to the HNF4α isoforms and LA metabolism – α7HMZ mice have greatly reduced levels of key cytochrome P450 genes that metabolize LA into bioactive oxylipins – *Cyp2c50*, *Cyp2c54* (75, 116). The significance of this finding remains to be determined.

In the end, the role, if any, of the HNF4α ligand in the transcriptional function of the protein remains to be elucidated. Nonetheless, one wonders whether it is simply a coincidence that LA is an essential fatty acid that every animal organism must obtain ultimately from plants and that HNF4 is just one of two nuclear receptors found in the oldest living animal organisms close to the time more than a billion years ago that animals diverged from plants and fungi (32, 117–119). Furthermore, one must consider the possibility that even if modern HNF4α is truly not functionally responsive to LA, it is possible that at some point during evolution LA (or some other ligand) did in fact act in a classical fashion. Perhaps as other nuclear receptors, such as the PPARs, evolved as long chain fatty acid binding transcription factors, HNF4 transitioned into a different mode of regulation. For example, more than 13 phosphosites were initially identified in HNF4α in absence of any sort of environmental cues (52), a number that has since more than doubled in Phosphosite Plus (120). Characterized phosphosites include that of AMPK (121, 122) which is activated in the fasted state, protein kinase C (PKC) (123) which has been shown to respond to polyunsaturated fatty acids like LA [e.g., (124, 125)] and Src tyrosine kinase which selectively targets P1-HNF4α but not P2-HNF4α (90). This selective activity of Src could explain the loss of P1-HNF4α and the retention of P2-HNF4α in both liver cancer and colon cancer – Src is known to be elevated in both (126).

## 13 Discussion

**Figure 6:**
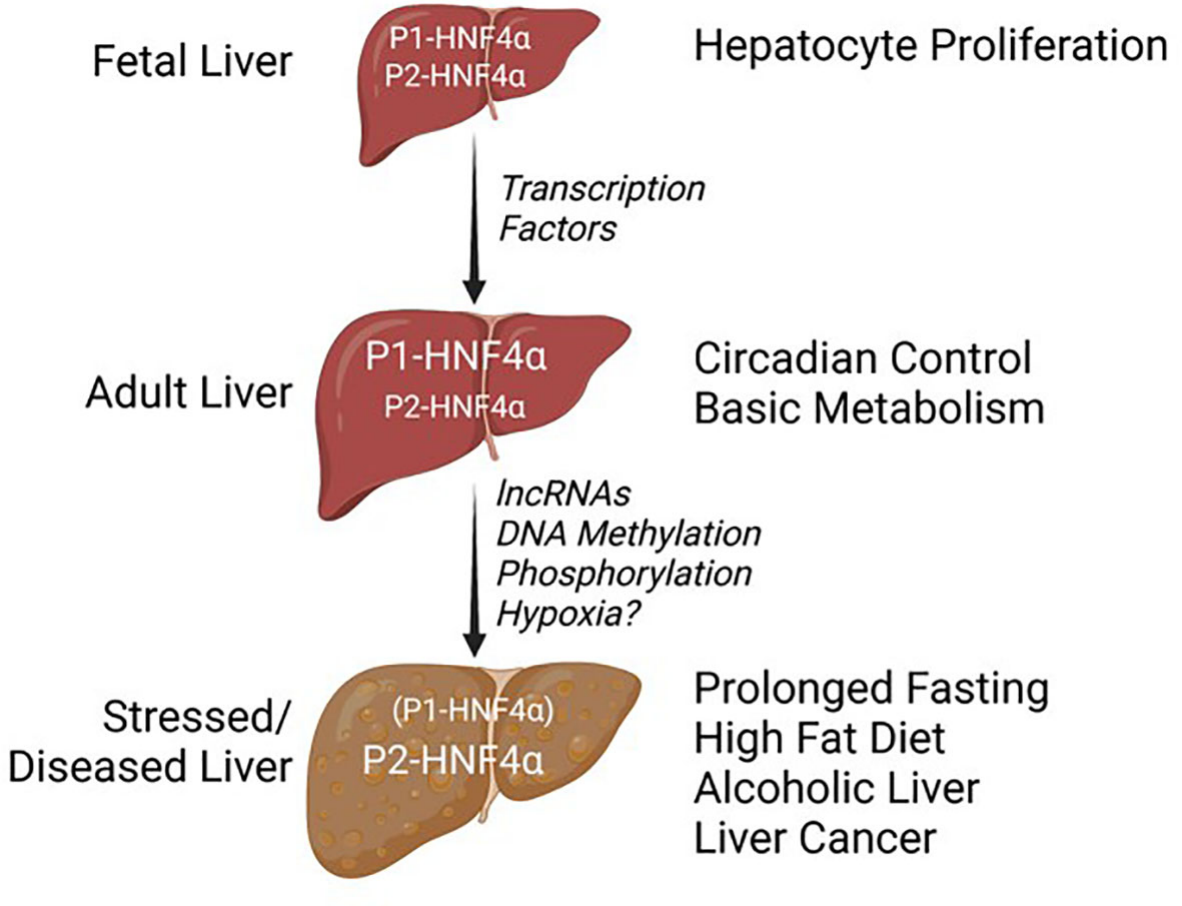
Balance of HNF4a isoforms in different stages of liver development and disease. See text for details.

In conclusion, many questions remain about what are the most critical functions of the P1- versus P2-driven HNF4α isoforms and why this dual promoter system has been conserved across so many species (**Figure 3C**). This is particularly relevant given that the exon swap mice expressing either only P1-HNF4α or P2-HNF4α are viable and healthy, unless they are subjugated to certain stresses. For example, α7HMZ mice, which express only P2-HNF4α, cannot survive a prolonged fast as well as WT or even α1HMZ mice (75); they are also extremely sensitive to experimentally induced colitis (78). P1- and P2-HNF4α are both expressed in the fetal liver but in a healthy, unstressed adult liver it is primarily the P1 promoter that is active as P1-HNF4α protein represses the P2 promoter (**Figure 6**). In the adult liver, HNF4α coordinates the expression of genes responsible for basic metabolism in conjunction with the circadian clock machinery, with P2-HNF4α being expressed only during limited times of the day/night. Certain metabolic stressors, including fasting, a high fat diet, alcoholic liver disease and liver cancer increase expression of P2-HNF4α by mechanisms that appear to involve promoter regulation by transcription factors, long ncRNAs and/or DNA methylation. Signaling molecules such as kinases could also impact the delicate balance of P1- and P2-HNF4α proteins.

While many mutations leading to MODY1 have been found in the P2-promoter, many fewer have been found in the P1-promoter (6, 127). This led to the assumption that MODY1 mutations were relevant primarily in the pancreas, where P2-HNF4α expression is dominant. Knowing now that P2-HNF4α is expressed in the adult liver under certain conditions of stress – including fasting and type 2 diabetes – raises the possibility that the effects of the MODY1 mutations in the P2 promoter could be due, at least in part, to an inability to express P2-HNF4α in the liver under key conditions (Da 83). Indeed, recent clinical findings suggest that certain MODY1 mutations in the coding regions may have an effect in the liver and kidney as well as the pancreas (11).

Similarly, the exact role of the HNF4α isoforms in liver cancer is not completely clear. A knockout of HNF4α increases chemically induced liver cancer in rodents and P1-HNF4α interacts with cyclin D1 in a negative reciprocal regulatory axis to control hepatocyte proliferation (4, 128, 129). But why is P2-HNF4α increased in liver cancer? Given that P1-HNF4α has been shown to repress the P2 promoter either directly or indirectly (80, 83, 104), could it be simply that the tumor suppressive P1-HNF4α must be decreased in order for the hepatocytes to proliferate and that once its expression is reduced, P2-HNF4α expression is coincidentally increased? It will be of interest to determine whether the negative regulatory loop between P1-HNF4α and cyclin D1 pertains to P2-HNF4α as well.

Finally, it is intriguing to speculate that differential expression of P1- and P2-HNF4α in the different zones of the liver could be involved in different metabolic functions such as ketogenesis versus gluconeogenesis. Hypoxic conditions near the central vein are associated with ketogenesis while normoxia is in the zone where gluconeogenesis occurs (**Figure 1**). HNF4α has been shown to associate with hypoxia inducible factor (HIF) in the kidney to turn on the expression of the erythropoietin gene (*EPO*) which stimulates red blood cell production (130): that was presumably P1-HNF4α, the only promoter known to be active in the kidney (34). In contrast, in pancreatic cells *in vitro* hypoxia activates AMPK which in turn decreases expression of HNF4α, presumably P2, by some as yet unknown mechanism (131). Clearly, many questions remain about the HNF4α isoforms in the liver, the genes they regulate, and the factors/conditions that regulate them. The next 30 years of HNF4α research will hopefully answer those and other questions that have not yet been formulated.

## Author contributions

All authors were responsible for conceiving, drafting, and critically revising this work, and were accountable for the accuracy and integrity of the work. All authors contributed to the article and approved the submitted version.
